# Single nucleotide polymorphism profile for quantitative trait nucleotide in populations with small effective size and its impact on mapping and genomic predictions

**DOI:** 10.1093/genetics/iyae103

**Published:** 2024-06-24

**Authors:** Ivan Pocrnic, Daniela Lourenco, Ignacy Misztal

**Affiliations:** Department of Animal and Dairy Science, University of Georgia, Athens, GA 30602, USA; Department of Animal and Dairy Science, University of Georgia, Athens, GA 30602, USA; Department of Animal and Dairy Science, University of Georgia, Athens, GA 30602, USA

**Keywords:** QTN, genomic prediction, ssGBLUP, GWAS, causative SNP, GBLUP, sequence, variants

## Abstract

Increasing SNP density by incorporating sequence information only marginally increases prediction accuracies of breeding values in livestock. To find out why, we used statistical models and simulations to investigate the shape of distribution of estimated SNP effects (a profile) around quantitative trait nucleotides (QTNs) in populations with a small effective population size (Ne). A QTN profile created by averaging SNP effects around each QTN was similar to the shape of expected pairwise linkage disequilibrium (PLD) based on Ne and genetic distance between SNP, with a distinct peak for the QTN. Populations with smaller Ne showed lower but wider QTN profiles. However, adding more genotyped individuals with phenotypes dragged the profile closer to the QTN. The QTN profile was higher and narrower for populations with larger compared to smaller Ne. Assuming the PLD curve for the QTN profile, 80% of the additive genetic variance explained by each QTN was contained in ± 1/Ne Morgan interval around the QTN, corresponding to 2 Mb in cattle and 5 Mb in pigs and chickens. With such large intervals, identifying QTN is difficult even if all of them are in the data and the assumed genetic architecture is simplistic. Additional complexity in QTN detection arises from confounding of QTN profiles with signals due to relationships, overlapping profiles with closely spaced QTN, and spurious signals. However, small Ne allows for accurate predictions with large data even without QTN identification because QTNs are accounted for by QTN profiles if SNP density is sufficient to saturate the segments.

## Introduction

Sequence data brings the opportunity to search for and use causative variants [quantitative trait nucleotides (QTNs)] for genomic predictions in livestock and plant breeding or the prediction of human polygenic risk scores. If most of the QTNs are known, predictions would be more accurate and persistent. So far, the use of putative QTN in genomic predictions has sometimes resulted in slightly increased accuracy but not always—e.g. see a review by Hayes and Daetwyler (2019). An increase of up to 5% accuracy was reported in the single-breed cattle population when preselected variants (close to putative QTN) from sequence data were added to the routinely used 60k SNP chip (VanRaden *et al.* 2017).

Potential QTNs or SNP close to QTN that possibly can be used to improve genomic predictions, e.g. as in Tiezzi and Maltecca 2015 for livestock or Izquierdo *et al*. 2024 for plants, are typically identified by genome-wide association studies (GWAS). For a general overview of the most recent developments and prospects in GWAS, see, for example, Visscher *et al*. (2017) and Abdellaoui *et al*. (2023) for human perspective, Cortes *et al*. (2021) for plant perspective, Johnsson and Jungnickel (2021) and Ros-Freixedes (2024) for livestock perspective, and Yáñez et al. (2023) for aquaculture species perspective.

A standard tool for traditional GWAS is a model where one marker is analyzed at a time as a fixed effect, whereas the polygenic effect is accounted for by fitting a relationship matrix among individuals under mixed models (Kennedy *et al.* 1992). Such a model is also known as efficient mixed-model association expedited (EMMAX) (Kang *et al.* 2010). Fitting a relationship matrix based on pedigree or genotypes reduces spurious signals due to population structure because it assumes individuals may share a considerable proportion of genes (Kennedy 1991; Kang *et al.* 2010). Alternatively, many recent studies are adopting Bayesian regression methods like BayesB (Meuwissen *et al.* 2001) or BayesR (Erbe *et al.* 2012) that consider all SNP jointly as random effects and estimate the effect of a SNP conditionally to all the other SNP. The SNP with larger signals are considered markers to nearby QTN. While the single SNP models use *P*-values to determine SNP significance, the joint SNP models usually estimate fractions of explained variance per segment of the genome, e.g. 1 Mb, and may use power and false discovery rates as arbitrary approaches to compare methods. While the golden standard for putative QTN is ≥1% of explained additive genetic variance (e.g. Chen *et al.* 2017), the origin of a 1 Mb segment is not clear.

Another GWAS method gaining momentum in livestock and plant populations is the single-step GWAS (ssGWAS) (Wang *et al.* 2012; Aguilar *et al.* 2019). This method estimates all SNP simultaneously, provides variance explained by each SNP together with a significance test (i.e. *P*-values equivalent to EMMAX; Duarte *et al*. 2014; Aguilar *et al*. 2019), and is based on single-step genomic best linear unbiased prediction (ssGBLUP) (Aguilar *et al.* 2010; Christensen and Lund 2010), which allows using information on all individuals concurrently, independently of the pedigree, phenotyping, and genotyping status. ssGBLUP is the standard tool for genomic predictions in livestock populations (Tsuruta *et al.* 2011; Christensen *et al.* 2014; Lourenco *et al.* 2015) and was recently applied to the UK Biobank data (Truong *et al.* 2020). Compared to other methods, ssGWAS allows using all available data and complete models (including multitrait models), potentially reducing spurious signals due to unaccounted selection or effects.

Independently of the method, an essential question for identifying QTN in livestock and plant populations is how the smaller effective population sizes limit the resolution of GWAS compared to humans. The genome comprises blocks or chromosome segments inherited from founders, separated by junctions (Fisher 1949, 1954); those junctions are swapping spots for the founder origin of segments. For randomly mating populations of constant size, the number of junctions is a function of effective population size (Ne) and genome length (*L*) (Stam 1980). Changing physical (not effective) population size (i.e. population growth, division, or bottleneck) strongly affects the number of junctions in small but not large populations (Chapman and Thompson 2002). Junctions define the genome segments; therefore, the inheritance is by segments, not by individual genes or QTN. A limited number of segments have important implications in GWAS as the segment size affects the resolution (Berisa and Pickrell 2016). For instance, Wang *et al.* (2012) found that the correlation between the effects of QTN and one adjacent SNP was lower than between a segment of 16 adjacent SNP (so-called windows) and QTN. Assuming a genome size of 3 Gb and the number of junctions of 10,000 in animals or 1 million in humans, the segment size and, subsequently, resolution of GWAS would be ∼approximately 300 kb in animals and 3 kb in humans. GWAS results provide evidence of this limited resolution. The Manhattan plots on individual SNP are usually noisy, and a common strategy is to smooth out noise by combining variance explained by SNP segments of, say, 1 Mb (Funkhouser *et al.* 2020). A limited number of segments constrain the dimensionality of genomic information for populations with small Ne and allow for high prediction accuracy of the genomic merit even with fewer data (Pocrnic *et al.* 2019).

While the above studies suggest that Ne limits the resolution of GWAS, it is possible to envisage a scenario where identifying QTN is feasible despite limited Ne. Assume a small number of QTN, all present in the data, a large number of phenotypic records, and an additive QTN model. If only QTN were used in SNP-BLUP or GBLUP-based models, the prediction accuracy would be close to 100%, as found by Fragomeni *et al.* (2017) or Pérez-Enciso *et al.* (2015). Then, QTN could be determined by an exhaustive search for the smallest set of SNP that results in almost 100% predictive accuracy. This scenario is hypothetical as most traits are likely to be controlled by many QTNs, with most below the detection threshold.

In this paper, we study the pattern of SNP distribution around a QTN in populations with varying Ne and numbers of genotyped individuals with phenotypes. This pattern is referred to as the QTN profile and helps understand the resolution of GWAS and its impact on genomic selection. Investigating the QTN profile around a QTN in GWAS and determining whether it is a function of effective population size likely require large datasets and many replicates. In this study, we determine the profile of QTN using data simulated to minimize the sampling variance. We also discuss the implications of this profile in methods used for genomic predictions.

## Materials and methods

### Chromosome segments and pairwise linkage disequilibrium

**Fig. 1.**
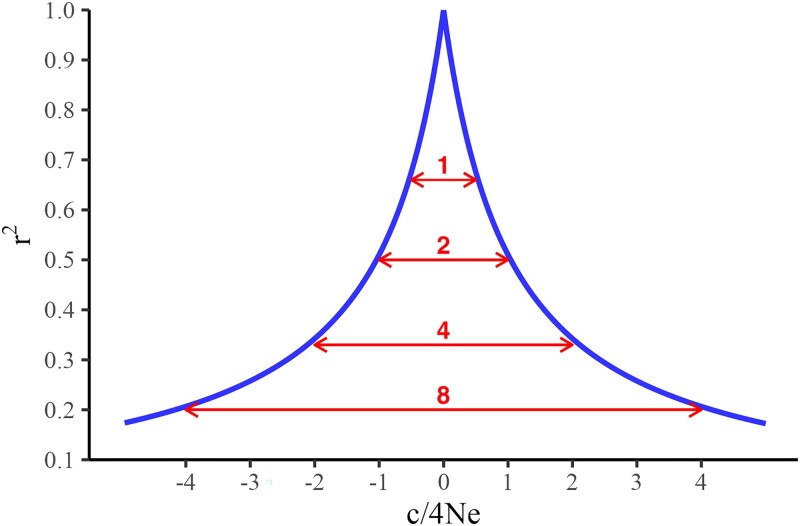
The expected value of PLD (*r*^2^) as a function of distance from the QTN in Stam segments.

The expected number of chromosome segments given by Stam (1980) is 4NeL, where Ne is the effective population size and *L* is the genome length in Morgans. Subsequently, the average size of a Stam segment (*s*) is 1/(4Ne) Morgan. Additionally, the expected pairwise linkage disequilibrium (PLD), represented by *r*^2^, was defined by Sved (1971) as


$$E(r^{2})=1/(4c\mathrm{Ne}+1),$$

where *c* is the genetic distance between two SNP in Morgans. The plot of *r*^2^ as a function of *c* in terms of Stam segments is given in Fig. 1. The interval where E(*r*^2^) declines to 0.67, 0.50, 0.33, and 0.20 corresponds to 1, 2, 4, and 8 Stam segments, as shown in Appendix. For more discussions on LD and chromosome segments, see Goddard and Meuwissen (2005).

### Phenotypic and genotypic data

Data for this study were simulated using the AlphaSimR package (Gaynor *et al.* 2021) and run using the R version 3.4.4 (R Core Team). Historical population genomes were generated using the Markovian coalescent simulator (MaCS) (CHEN *et al*. 2009) as implemented in the package. We used the default options of the coalescent simulator via the “runMacs2” function in the AlphaSimR package, except we fixed the base population Ne to either 60 or 600 (depending on the scenario), gradually decreasing from Ne of 100,000 about 1 million generations ago (de ROOS *et al*. 2008). In all scenarios, the simulated genome had 10 chromosomes with equal lengths of 100 cM each. The recombination rate was set to 1.0 × 10^−8^, and the mutation rate was set to 2.5 × 10^−8^. To limit the computations, 50,000 biallelic SNP markers were generated, equally spaced along the chromosomes, resulting in 50 SNP per cM. Equal placement of genetic markers was achieved by modifying the default genetic map in AlphaSimR. Each chromosome harbored 10 QTNs that were assigned the same additive effect and placed in the same locations across the 10 chromosomes, corresponding to the locations of actual SNP markers. The simulated QTN additive effects slightly differed between the scenarios and were approximately 0.25, 0.22, and 0.24 for NE60, NE60_3x, and NE600, respectively. The QTNs were separated by at least 500 SNP (≈10 cM) to reduce interference.

The first recent generation (base population) was created using 6,000 individuals from the historical population, followed by nine generations of random mating to ensure that the Ne remained as close as possible to that simulated in the historical population. In the simulation of those nine generations, we used the same recombination rate but assumed no mutations. This is a common limitation in many simulation studies, as incorporating a realistic level of mutation into gene drop simulation of the whole genomes is complex. For example, efficient methods for simulating such mutations are currently being developed (Baumdicker *et al.* 2022). The number of mating males and females per generation was set to 15 and 1,000 for Ne = 60, 175 and 1,000 for Ne = 600, and 15 and 3,000 for Ne = 60, but with three times more individuals per generation. Further on, these scenarios will be referenced as NE60, NE600, and NE60_3x, respectively.

Two progenies of equal sex ratio were created per mating, resulting in either 2,000 (NE60 and NE600) or 6,000 (NE60_3x) individuals per generation. Phenotypes for a quantitative trait were generated assuming a heritability of 0.5 and with a single record per individual; a large heritability allows for clear results with less data. Pedigree and phenotypes were recorded for all 24,000 (NE60 and NE600) or 60,000 (NE60_3x) individuals across 10 generations. Genomic information was available for the last three generations, i.e. 6,000 (NE60 and NE600) or 18,000 (NE60_3x) genotyped individuals.

### Single-step genome-wide association analysis

Most of the GWAS methods assume that genotypes and phenotypes are available in the same individuals; however, in livestock populations, those two sources of information may be available in different sets of individuals. Because of that, we used ssGWAS (Wang *et al.* 2012; Aguilar *et al.* 2019) under the following model:


$$y=1\mu +Wu+e,\;\;\;\;\mathrm{Var}(e)=I\sigma_{e}^{2},\;\mathrm{and}\;\;\mathrm{Var}(u)=H\sigma_{u}^{2}$$

where ***y*** is a vector of phenotypes, *μ* is an overall mean, **u** is a vector of random additive genetic effects, **e** is a vector of random residuals, **W** is an incidence matrix relating observations in **y** to additive genetic effects in **u**, **H** is a realized relationship matrix, $\sigma_{u}^{2}$ is the additive variance, and $\sigma_{e}^{2}$ is the residual variance. Variance components were assumed to be known using the base population simulation parameters; $\sigma_{u}^{2}=\sigma_{e}^{2}=1.00$. The realized relationship matrix **H** combines pedigree and genomic relationships, with the inverse as in Aguilar *et al.* (2010):


$$H^{-1}=A^{-1}+\left[\begin{array}{cc} 0 & 0\\ 0 & G_{b}^{-1}-A_{22}^{-1} \end{array}\right],$$

where **A** is the pedigree-based numerator relationship matrix for all individuals included in the analysis and **A**_22_ is the pedigree-based numerator relationship matrix for genotyped individuals. To ensure the matrix was invertible, the initial **G** was blended prior to inversion as $G_{b}=\alpha G+(1-\alpha )A_{22}$, with α = 0.95, and the initial **G** defined as in VanRaden (2008):


$$G=\frac{ZDZ^{\prime }}{2\Sigma p_{j}(1-p_{j})},$$

where Z is a matrix of allele content centered for allele frequencies, $p_{j}$ is the allele frequency for marker *j* in the current genotyped population, and **D** is a diagonal matrix of weights for SNP markers. All SNP were assumed to have equal weight; therefore, **D** was an identity matrix (**I**). After computing genomic estimated breeding values, SNP effects (**a**) were obtained as in Wang *et al.* (2012):


(1) $$\hat{a}|\hat{u}=\;\lambda \alpha \delta {DZ^{\prime }G}_{b}^{-1}\hat{u},$$

where *λ* is a ratio of SNP to additive variance used in the data simulation, δ=(1−ρ/2), and *ρ* is the average difference between all elements of G and $A_{22}$, which is known as the tuning parameter used to adjust the genetic base of G to $A_{22}$ (Vitezica *et al.* 2011). The *P*-values for SNP were computed based on Aguilar *et al.* (2019):


$$P\text{-}\mathrm{valu}e_{i}=2\left(1-\Phi \left(\left|\frac{{\hat{a}}_{i}}{\mathrm{sd}({\hat{a}}_{i})}\right|\right)\right),$$

with Φ being the cumulative standard normal function and $\mathrm{sd}({\hat{a}}_{i})$ the square root of prediction error variance (PEV) of the *i*th SNP effect. Prediction error variance for each SNP effect was:


(2) $$\mathrm{Var}({\hat{a}}_{i})=\lambda \alpha \delta \;z_{i}^{\prime }G_{b}^{-1}(G_{b}\sigma_{u}^{2}-C^{u_{2}u_{2}})G_{b}^{-1}z_{i}\;\delta \alpha \lambda .$$

All SNP that passed the Bonferroni threshold of 10^−6^ were considered statistically associated with the simulated trait. Computations were performed by the BLUPF90 software suite (Misztal *et al.* 2014).

### Pooled SNP effects for QTN profiling

The QTN and SNP were simulated at the same positions across the 10 chromosomes, so to assess the QTN profiles, we have averaged the effects of 100 SNP with the same distance from the QTN across all QTNs. The averaging included 50 SNP upstream and downstream of the QTN. This is equivalent to averaging segments of approximately 1 cM for a population with Ne equal to 60.

## Results and discussion

In this study, we used the ssGWAS on the simulated datasets with varying effective population sizes and number of genotyped individuals with phenotypes. Our results indicate that more significant SNP are captured with a larger sample size or larger effective population size. Furthermore, we show that the width of the distribution of estimated SNP effects around QTN (i.e. the QTN profile) is a function of the effective population size, with the hypothesis that the PLD curve can be used to describe the underlying (real) QTN profile. To this end, we discuss qualitative results from the ssGWAS: (1) by inspecting the Manhattan plots; (2) by inspecting the pooled estimated SNP effects; and (3) by decomposing the contributions to the Manhattan plots into signals from the QTN itself, QTN profile, relationships, and noise. Finally, we discuss (4) potential gaps in the coalescent simulation from the viewpoint of generated LD and allele frequency spectrum and resemblance to real data, and we close the discussion with (5) implications for genomic predictions.

### (1) Genome-wide manhattan plots

**Fig. 2.**
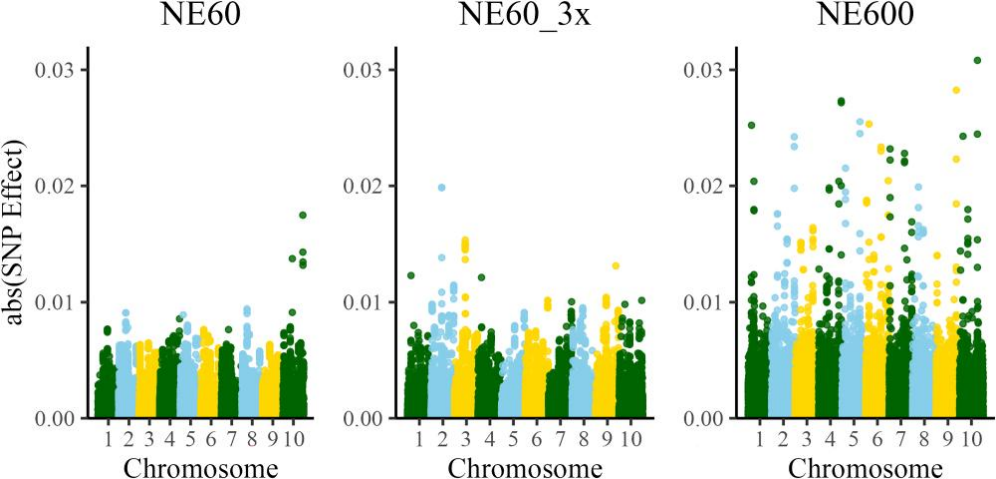
Manhattan plots for SNP effects computed for datasets with effective population size 60 (NE60), with the same effective population size but three times more data (NE60_3x), and with effective population size 600 (NE600).

The Manhattan plots of estimated SNP effects ($\mathrm{abs}({\hat{a}}_{i})$) for NE60, NE60_3x, and NE600 are shown in Fig. 2. Because 100 equidistant QTNs were simulated with identical effects, there was an expectation of observing roughly 100 similar peaks, conditional on their allele frequencies. However, only a few peaks with different values could be identified. Visually, the number of large signals increased from NE60 to NE600, with NE60_3x in between the two. The maximum estimated SNP effect (top peak in Manhattan plot) explained about 7% of the simulated QTN additive effect for NE60, about 9% for NE60_3x, and about 13% for NE600. The differences in signals from QTN could be due to changes in allele frequencies because of natural selection or drift, but since no directional selection was simulated and the mating was random, the main differences are mainly result of differences in initially simulated QTN allele frequencies as given by coalescent simulation. We discuss some properties of coalescent simulator in “Simulation assumptions and resemblance to real data” subsection. The minor allele frequencies for all simulated SNP markers and SNP markers where the QTNs are located are presented in Supplementary Files 1 and 2. Furthermore, we investigated the relationship between the minor allele frequencies of SNP markers where the QTNs are located and corresponding estimated SNP effects. As presented in Supplementary File 3 for the NE60 scenario, there was a general trend that higher minor allele frequencies corresponded to a larger estimated SNP effect, but the trend was not linear nor had a clear pattern. Similar observations were found across scenarios (not shown).

**Fig. 3.**
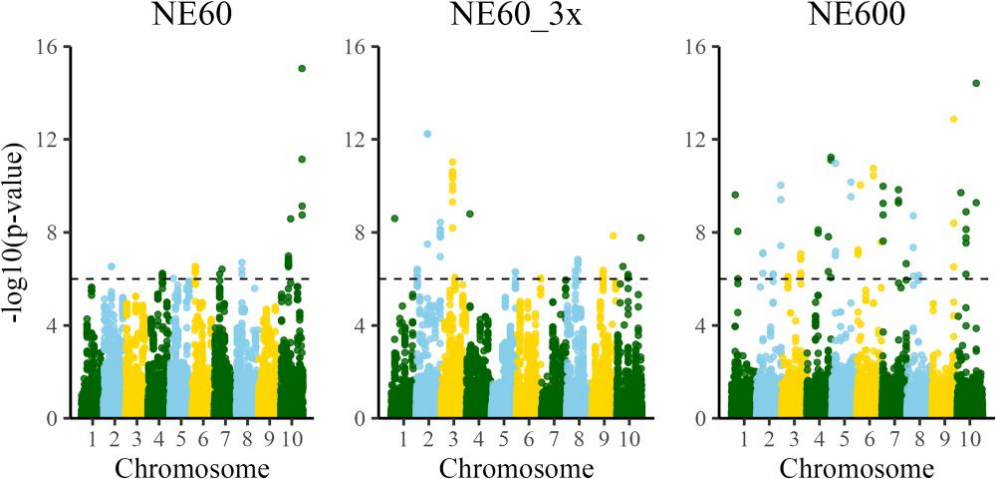
Manhattan plots for *P*-values computed for datasets with effective population size 60 (NE60), with the same effective population size but three times more data (NE60_3x), and with effective population size 600 (NE600). The dashed lines represent a significance threshold of 10^−6^.

To assign significance to the signals while accounting for population structure, we recreated the Manhattan plots for *P*-values using the scale of −log 10 (*P-*value), which are shown in Fig. 3. The number of significant SNP (*n*), i.e. the SNP above the threshold of 6 on the −log 10 scale, was the smallest with NE60 (*n* = 38), larger with NE60_3x (*n* = 57), and the largest with NE600 (*n* = 64). Figures 2 and 3 were comparable, indicating that the plots based on SNP effects and *P*-values are visually similar (Supplementary File 4 shows them grouped by scenarios). While Fig. 2 is based on the value of the SNP effect, Fig. 3 is based on a function of the SNP effect adjusted for its individual SD. The plots are approximately proportional when the SDs of all SNP are similar, although not linearly. More significant SNP were captured with a larger sample size or larger Ne. The association signals are more clear with more individuals and, subsequently, with more recombination.

**Fig. 4.**
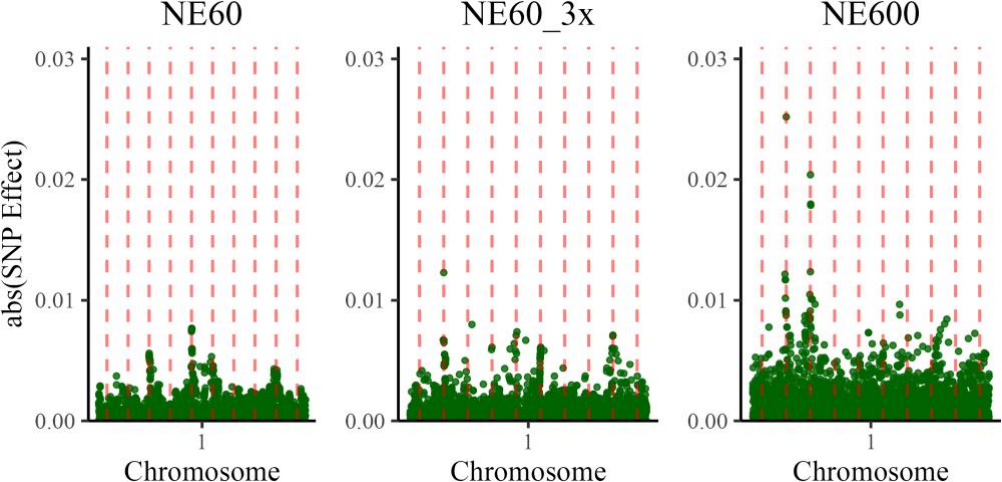
Manhattan plots for SNP effects—first chromosome only—computed for datasets with effective population size 60 (NE60), with the same effective population size but three times more data (NE60_3x), and with effective population size 600 (NE600). Dashed vertical lines indicate the QTN locations.

Since our aim was to visualize the QTN profile plots, we zoomed into the Manhattan plots for individual chromosomes. As an example, Fig. 4 shows the Manhattan plots of SNP effects from the first chromosome with locations of each of the 10 QTN shown as vertical dashed lines. Only a few of the 10 simulated QTNs (vertical dashed lines) had a trail of SNP although with a smaller magnitude than the simulated effects. Because of the high noise level, QTN profiles were not evident from these plots.

### (2) Pooled SNP effects and the QTN profile

**Fig. 5.**
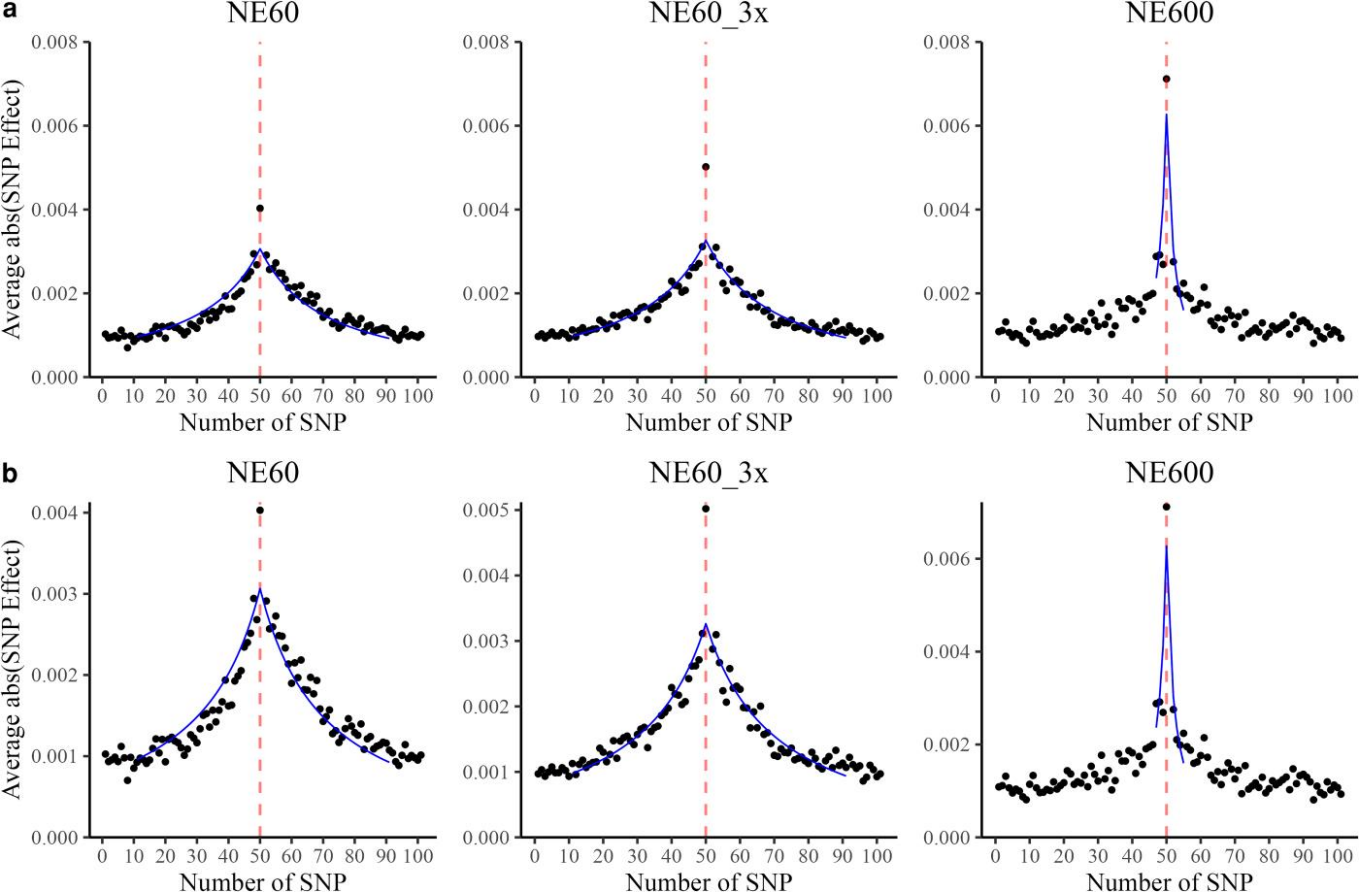
Profile of QTN or distribution of SNP around QTN (a—*y*-axis scaled to the maximum value of NE600 scenario and b—nonscaled *y*-axis), computed for datasets with effective population size 60 (NE60), with the same effective population size but three times more data (NE60_3x), and with effective population size 600 (NE600). Dashed vertical lines indicate locations of simulated QTN. Curves indicate the best fit of the PLD curve within ± 2 Stam segments; *R*^2^ for the fit excluding the QTN is 0.87, 0.85, and 0.78 for NE60, NE60_3x, and NE600, respectively.

As all QTNs were simulated with the same effect, it was possible to reduce the noise by averaging the effects of 50 SNP upstream and downstream from QTN, equivalent to averaging segments of approximately 1 cM for Ne equal to 60, as shown in Fig. 5. In all scenarios, the maximum peak response was at the true QTN position, with the remaining SNP showing a distribution with a sharp peak, similar to a Laplace distribution as in Bayesian models (de los Campos *et al.* 2009). For Ne 60, the averaged response had a similar distribution, although the variability around the curve was higher for scenario with less data (NE60), and the peak was more apparent for scenario with more data (NE60_3x). For Ne 600 (NE600), the average profile was lower and narrower, with the clear peak at the QTN. To assess whether the QTN allele frequency affected the profile plots, we recreated the plots with filtered QTN (20 QTN with the highest minor allele frequency) as presented in Supplementary File 5. With the QTN filtered based on the highest minor allele frequency, we got qualitatively similar QTN profiles with only difference in the scale. On the contrary, by filtering 20 QTNs with the lowest (close to zero) minor allele frequency the QTN profile and corresponding peak at the QTN were not apparent (not shown).

Above the value of 1 SD of average SNP effect (approximately between 0.001 and 0.002 in Fig. 5, depending on the scenario), the profile was about 60 SNP or 1 cM wide for NE60 and about 10 SNP or 0.2 cM wide for NE600. The five times wider profile with NE60 compared to NE600, despite the 10 times difference in Ne, could be due to ignoring the profile below the value of 1 SD where many points appear random. Using the formula by Stam (1980), the number of independent chromosome segments, equivalent to the number of genome segments, is 4NeL (where *L* is the genome length in Morgan) or 2,400 for Ne 60 and 24,000 for Ne 600. Assuming 50k SNP, this would correspond to a segment of approximately 20 SNP for Ne 60 and 2 SNP for Ne 600. A wider profile in GWAS than that of independent chromosome segments means that the profile spans many independent chromosome segments. The effective number of independent chromosome segments (typically abbreviated as Me) is a well-established concept in the literature, particularly in the context of genomic prediction accuracy. For an in-depth discussion on this topic, refer to studies by Daetwyler *et al*. (2008), Goddard (2009), Hayes *et al*. (2009), and Brard and Ricard (2015), among others.

We hypothesize that the QTN profile is a function of PLD (*r*^2^) as defined by Sved (1971): E(*r*^2^) = 1/(4*c*Ne + 1), where *c* is the genetic distance between two SNP, expressed in Morgans. Such a formula is visualized in Fig. 1, with numbers represented as the length of one segment as derived from the formula by Stam (1980), where one segment is 1/(4Ne) Morgans. The expected PLD decays to 0.67 for an interval of 1 Stam segment, 0.50 for an interval of 2 Stam segments, 0.33 for an interval of 4 Stam segments, 0.20 for an interval of 8 Stam segments, and 0.10 for 18 Stam segments. In this study, 1 Stam segment would be about 20 SNP for Ne equal to 60 and 2 SNP for Ne equal to 600. Subsequently, the PLD would decay to 0.33 for an interval of 80 SNP in the NE60 and NE60_3x scenarios and 8 SNP in the NE600 scenario. Assuming that PLD is the real QTN profile, SNP in 2 (4, 8) Stam segments would correspond to 50% (66%, 80%) of the total response to one QTN (see Appendix).

Figure 5 shows profile plots for the SNP effects fitted with the PLD curves, displaying similar shapes. For intervals of ± 2 Stam segments around the QTN (80 SNP for NE60 and NE60_3x and 8 SNP for NE600) and excluding the QTN, the fit was precise for NE60 (*R*^2^ = 0.87) and NE60_3x (*R*^2^ = 0.85) and somewhat less for NE600 (*R*^2^ = 0.78) scenario. A slightly poorer fit with NE60_3x compared to NE60 could be due to less shrinkage of the QTN effect. Less fit with NE600 is due to insufficient crossovers to saturate an 8 SNP interval. With 3k individuals and a genome length of 10 Morgans, there are only 30k crossovers, or approximately one every 2 SNP, insufficient for a good fit. Therefore, larger data and more SNP would be required to improve the fit with NE600.

### (3) Components of Manhattan plot

**Fig. 6.**
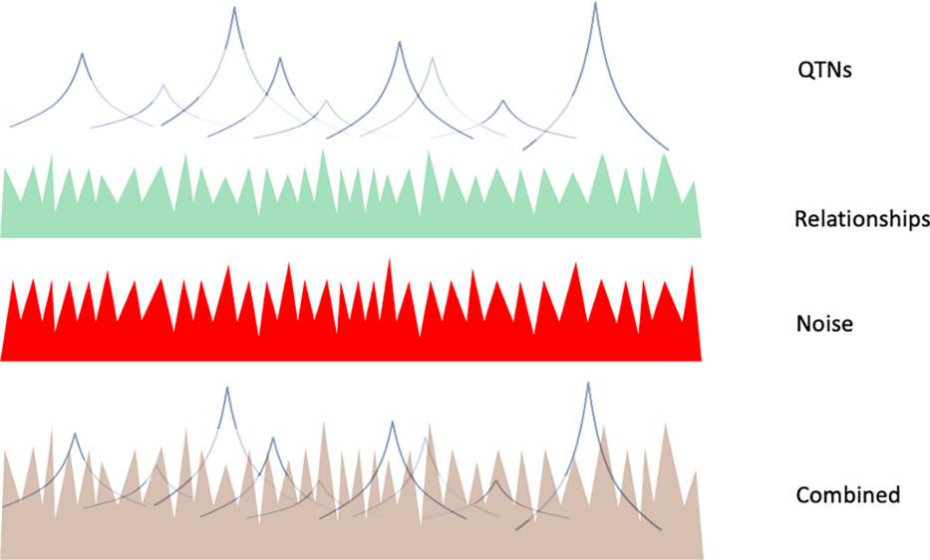
Components of a Manhattan plot and their composite plot for a small Ne and medium dataset. With larger data, the noise component will decrease, signals due to QTNs will increase, and signals due to relationships will decrease if signals due to QTNs explain a large fraction of the additive variance.

**Fig. 7.**
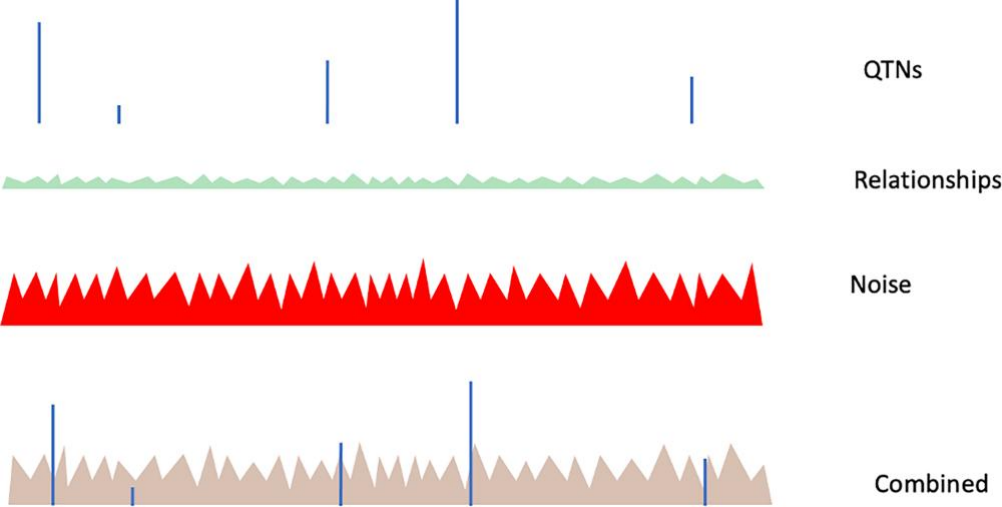
Components of a Manhattan plot and their composite plot for a large Ne. With larger data, the noise component will decrease, and signals due to QTNs will increase. Signals due to QTN and the relationships will be clear only with a sufficiently large number of SNP.

The predictive ability of GBLUP-based methods is mainly independent of the number of QTN (Lourenco *et al.* 2017; Takeda *et al.* 2021) and is attributed to exploiting differences between the expected and realized relationships (VanRaden 2008). SNP-BLUP, and indirectly GBLUP, partially account for QTN as shown in this study; however, the signals due to QTN are affected by shrinkage and noise, the latter partly due to estimation error, genotyping errors, and a small number of SNP. Assuming that PLD is a good predictor of QTN profiles, it is possible to identify components of the Manhattan plot, as illustrated in Figs. 6, and 7. The plots are composed of signals due to relationships, LD with QTN following the PLD curve, actual QTN (if present in the data), and signals due to noise because of the estimation error, a finite number of SNP, and a limited number of samples. The conceptualizations and patterns presented in Figs. 6, and 7 are arbitrary and meant to illustrate pseudo-random variation.

While accuracies of genomic relationships are high with a typical number of SNP (SD < 0.5% with 40k SNP as in VanRaden 2008), signals due to relationships appear as semi-random noise as defined by formula (1)when all QTNs are small, with predictor variance as in (2). Signals due to QTN and QTN profiles are visible only when they are large enough to rise above the signals due to relationships and noise. Signals due to LD with QTN are wide for populations with small Ne, with 4 Stam segments accounting for up to 66% of QTN variance and 8 Stam segments accounting for up to 80% variance (see Appendix). The fraction of the QTN variance explained by the segments depends on Ne, the amount of data, and the distribution of the QTN effects. As only a fraction of QTN with similar effects were observed in this study, there is a strong confounding of QTN signals with other signals.

Figure 6 illustrates the Manhattan plot for a medium dataset and small effective population size. With a small dataset, signals due to relationships prevail as signals due to QTN are small because of shrinkage, with a risk of pseudo-random peaks being interpreted as QTN or markers to QTN. In a study involving a fertility trait with low heritability in a small population with about 2,000 dairy cattle (Kiser *et al.* 2019), the Manhattan plots lacked resolution, and many SNP were labeled as causative variants. With a large dataset, the estimation error and shrinkage are smaller (Jang *et al.* 2023). When many large QTNs are present and now account for a large fraction of the additive variation, signals due to relationships decrease. When few large QTNs are present, and QTN profiles explain a small fraction of the additive variance, signals due to relationships remain the dominant part of the Manhattan plot. In a study involving a large population of 36,000 high-accuracy bulls (Jiang *et al.* 2019), the Manhattan plots were clear and showed many peaks with precise LD patterns.

Figure 7 illustrates the Manhattan plot for a population with a very large effective size, e.g. humans. Signals due to relationships are very small, QTN profiles are very narrow, and the Manhattan plot is mainly composed of estimation error and very narrow profiles of SNP. Assuming genome length of 30 Morgans, 8 Stam segments accounting for 80% of the QTN variance would be 2 Mb for a population with Ne 100 (e.g. cattle), 5 Mb for a population with Ne 40 (e.g. chicken and pigs), and only 20 kb for a population with Ne 10,000 (humans). When chosen experimentally by minimizing noise and maximizing information, the window size varied from 1 Mb in cattle (Buchanan *et al.* 2016) to 10 Mb in chicken (Stainton *et al.* 2017).

**Fig. 8.**
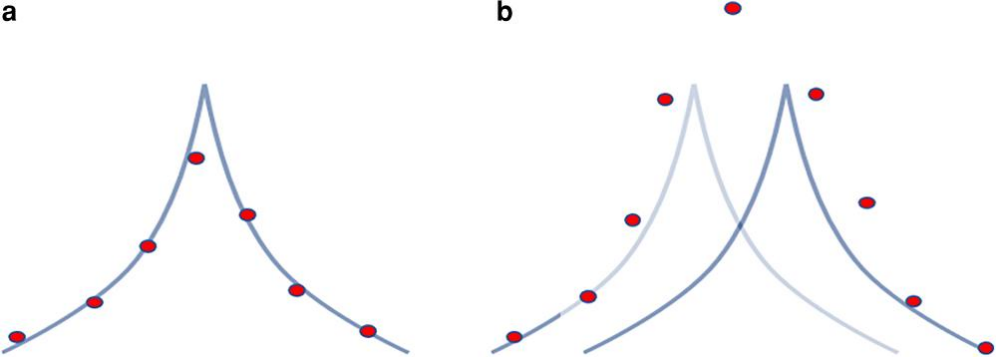
Accounting for QTN by GBLUP for single (a) QTN and two close (b) QTNs.

With very large datasets, GBLUP or SNP-BLUP incorporate large QTN by accounting for QTN profiles, as illustrated in Fig. 8 for a single QTN and for two close QTNs. While a SNP chip is not likely to contain QTN, it has enough SNP to cover the QTN profile, and the coverage can be pretty good with large data. With two close QTNs, the QTN profiles would overlap but would still be accounted by GBLUP. Wang et al. (2012) looked at the prediction of QTN effects in a simulation study, and the best estimates were not with the nearest SNP effect but with a sum of 16 nearby SNP, indicating the optimum window size of 16 SNP. With a small effective population size, QTN profiles of adjacent QTN are likely to overlap, and the observed peak in a Manhattan plot may be a composite of many QTN (see Fig. 8). The ability of GBLUP to account for QTN is a very valuable outcome for commercial genetic evaluations in plants and livestock. Most models used for the genetic evaluation are multitrait, and accounting for different QTNs for each trait would lead to excessive computations (Tiezzi and Maltecca 2015).

### (4) Simulation assumptions and resemblance to real data

In our study, we used very strong assumptions to visualize the profile of QTN, including equal allele substitution effects for QTN, a small number of evenly spaced QTN, and equal recombination rate across the genome. In reality, most traits are complex and, therefore, controlled by a large number of not evenly spaced QTN, with only a few having a large effect size. After intensive selection, most genes with a large effect become fixed, and large distributing genes are not fixed mainly because of pleiotropy, as documented by Georges *et al.* (2019). In the end, the only QTN of interest would be the top QTN not showing excessive pleiotropy. While this study looked at a single trait only, determining pleiotropy requires multitrait GWAS. In a study of two populations of pigs (Wientjes *et al*. 2023), several significant peaks were found for many traits. However, no peaks were found in a composite index of these traits, indicating pleiotropy for all the QTNs associated with the peaks. Generalizing, top QTN could be of little interest commercially for long selected species, although these QTN may be useful in species starting the selection, e.g. in fish.

In the simulation, the number of chromosome segments computed as 4 NeL (Stam 1980) was 2,400 for NE60 and NE60_3x, and 24,000 for NE600. Assuming that 12 SNP per segment are needed to identify most junctions between segments in the population (Macleod *et al*. 2005; Pocrnic *et al*. 2016), the minimum number of SNP would be about 30k for NE60 and NE60_3x and 300k for NE600. Thus, the simulation had enough SNP for NE60 but too few for NE600. For human studies, the number of segments is around 1.2 M, and identification of most junctions would require a 15 M SNP chip.

Our genomic data generation process relies on the sequentially Markovian coalescent model as implemented in MaCS (Chen *et al*. 2009). In this sense, our results could be impacted from two angles: unrealistic LD pattern and unrealistic allele frequency spectrum. Firstly, such models could result in unrealistic LD pattern, particularly long-range LD, especially when sampling many individuals relative to the effective population size, thus impacting the size of the segments in our study (Nelson *et al.* 2020). While normally this leads to QTN profiles that are too narrow, the nine generations of random mating that are used following the coalescent simulation were sufficient to compensate at least qualitatively. Secondly, the ascertainment bias plays a role in the realized allele frequency spectrum, as typically found across studies using simulated data, but it is hard to quantify its impact. The coalescent simulators are efficient in simulating neutral variation with gradually smaller Ne to mimic drift and selection due to domestication and recent selective breeding. This creates variation with many rare variants and a typical U-shaped allele frequency spectrum (Daetwyler *et al.* 2013). The whole-genome sequencing in real populations can capture rare variants, while SNP arrays do not, and thus have uniform allele frequency spectrum due to the SNP ascertainment bias, which can lead to a mismatch between simulated and real data. Nevertheless, quantifying the impact of these assumptions on the accuracy of GWAS and genomic predictions, as well as on genetic variance, is challenging. The current literature suggests that the impact on accuracy is limited since many livestock breeding populations have high levels of linkage disequilibrium (e.g. Daetwyler *et al.* 2013; Hickey *et al.* 2013), but more research is needed in this field.

### (5) Implications for genomic predictions

This study raises a question on the optimal SNP selection (or weighting of a genomic relationship matrix) based on statistical criteria applied by common methods, and its impact on the accuracy of genomic predictions. Selected SNP can either be the actual QTN, markers to QTN as QTN profiles, markers due to relationship signals, or due to noise. The success of SNP selection also depends on the genetic architecture of traits (Zhang *et al.* 2016). With a few QTN, all of them can be identified and estimated well for high prediction accuracy. With many QTN, selected SNP would likely include only a few QTNs (Fragomeni *et al.* 2017). Brøndum *et al.* (2015) stated that aside from knowing the variance of the QTN, knowing their positions helps to assign the variance to the correct variant, avoiding either shrinkage or inflation; shrinkage is less important with few SNP.

Signals due to relationships are weak in a population with a very big Ne, and QTN profiles are narrow. Then, the only choice for high accuracy is the identification of QTN or markers that are very close to QTN. Such an identification would require a very large SNP chip so that the individual SNP would fall within narrow QTN profiles or sequence information. For populations with small effective population size, signals due to relationships would be strong, QTN profiles would be wide, the number of QTN with large effect would be small except in simulation studies or for unselected traits, and the identification of actual QTN would be hard. With a small dataset, SNP selection may increase the accuracy of predictions due to the reduction in the dimensionality of the genomic information even if the selected SNP are mostly due to signals due to relationships (Karaman *et al.* 2016; Lourenco *et al.* 2017; Pocrnic *et al.* 2019). With very large phenotypic data, when signals due to noise would be small, large accuracy can be obtained without QTN identification since QTN can be accounted via QTN profiles. With medium datasets, the accuracy with SNP selection would be somewhat higher if the largest QTN or their markers can be identified; identification of actual SNP would require a sequence data.

A study by Fragomeni *et al*. (2019) provides a glimpse into factors affecting accuracy with sequence information. Reliabilities (squares of accuracy) were calculated for stature in Holsteins, where the genomic information included 54k generic SNP and 17k putative QTN on 27k bulls. Additionally, phenotypic information was available on 3 M cows. Initial analyses by GBLUP used pseudo-observations on the bulls. The base reliability with unselected 54k SNP was 69%, increased to 70% after including 17k putative SNP, and increased again to 71% with weighting the genomic relationship matrix; weighting is a form of SNP selection. After correcting the model for a different amount of information per bull, the reliabilities increased to 73%, with no advantage for weighting. After changing the model to ssGBLUP, where the phenotypes of cows were modeled directly, the reliability increased to 76%, again with no advantage for weighting. The study illustrates the point that an improved chip may improve the accuracy (1% in this case), SNP selection or weighting may compensate for an inferior model, and better modeling with more data has a much higher impact.

One of the goals of the 1,000 bull genomes project was finding QTN based on sequence data, acknowledging that SNP in the regular chips (i.e. from 10k to 777k) are insufficient to capture the information about QTN (Hayes and Daetwyler 2019). Therefore, the central hypothesis behind discovering and using QTN in genomic evaluations is to maximize prediction accuracies. However, the reported gains from sequence variants are only marginal (e.g. Veerkamp *et al.* 2016; Jang *et al*. 2022; Ros-Freixedes *et al.* 2022). Summarizing earlier developments, small gains are likely for several reasons: the inability to identify the true causative QTN due to the wide QTN profiles in livestock populations, few large QTNs existing in selected populations, pleiotropy, and GBLUP increasingly accounting for QTN with larger data.

### Conclusions

The Manhattan plots are composed of signals from QTN, LD to QTN called QTN profile, relationships, and noise. The QTN profile is similar in shape to a PLD curve and has a width inversely proportional to the effective population size. With large effective population size, QTN profiles are narrow, relationships are weaker, and QTN identification is relatively easy with large phenotypic data. With a small effective population, signals due to QTN profiles are wide and confounded with strong signals due to relationships, resulting in limited resolution of GWAS and poor discovery rate. Genomic prediction in populations with large effective population size requires high-density SNP and identification of QTN or markers close to QTN. Genomic prediction in populations with small effective population size is sufficiently accurate with medium-density SNP, and with large data, they account for QTN via QTN profiles, even without the actual QTN identification. QTN profiles justify showing Manhattan plots as a percentage of variance explained in moving windows. In such a case, the optimal window size for a population with Ne 100 is 1–2 Mb wide, and for a population with Ne 1,000, it would be 0.1–0.2 Mb wide.

## Supplementary Material

iyae103_Supplementary_Data

## Data Availability

The simulated data underlying this article (SNP genotypes, phenotypes, pedigrees, simulation, and analysis parameter files) are available in the Zenodo open research repository at https://doi.org/10.5281/zenodo.10798081. Otherwise, the authors state that the data necessary to confirm the conclusions presented in the article are represented fully within the article. Supplemental material available at GENETICS online.

## Appendix

### PLD curve and Stam segments

The expectation of PLD (*r*^2^) as a function of effective population size (Ne) and distance from QTN (in Morgans), represented by *c*, was quantified by Sved (1971) as:

$$E(r^{2})=1/(4c\mathrm{Ne}+1)$$

Assuming one Stam segment as 1/(4Ne), the curve can be rewritten as

$$E(r^{2})=1/(n+1)$$

where *n* is the distance from the QTN in Stam segments. Thus, *r*^2^ declines to 0.67 for an interval of 1 Stam segment, 0.50 for an interval of 2 Stam segments, and 0.20 for an interval of 8 Stam segments.

Assume *q* SNP per Stam segment, with the QTN represented by SNP 0. Assuming that the SNP value due to a single QTN is proportional to PLD,

$$a_{i}∼\frac{1}{1+i/q}$$

where *q* is a constant and the variance assuming equal gene frequency is $\;∼\;a_{i}^{2}$. Then, the fraction of a QTN variance accounted for within *t* Stam segments is

$$\frac{\sum_{i\;=\;-tq}^{tq}\;{\left(\frac{1}{1+i/q}\right)}^{2}}{\sum_{i\;=\;-\infty }^{\infty }\;{\left(\frac{1}{1+i/q}\right)}^{2}}$$

Numerical computations show that the interval of 2 segments explains about 50% of the QTN variance, 4 segments explain 66%, and 8 segments explain 80%.
