# Supplementary material for: Single nucleotide polymorphism profile for quantitative trait nucleotide in populations with small effective size and its impact on mapping and genomic predictions
Source: Genetics. 2024 Jun 24;227(4):iyae103. doi: 10.1093/genetics/iyae103 (PMC11304960; doi:10.1093/genetics/iyae103)

**Supplemental File 2.** Minor allele frequencies for simulated SNP markers that harbor QTN calculated based on the individuals from the last three generations for datasets with effective population size 60 (NE60), with the same effective population size but 3 times more data (NE60_3x), and with effective population size 600 (NE600).


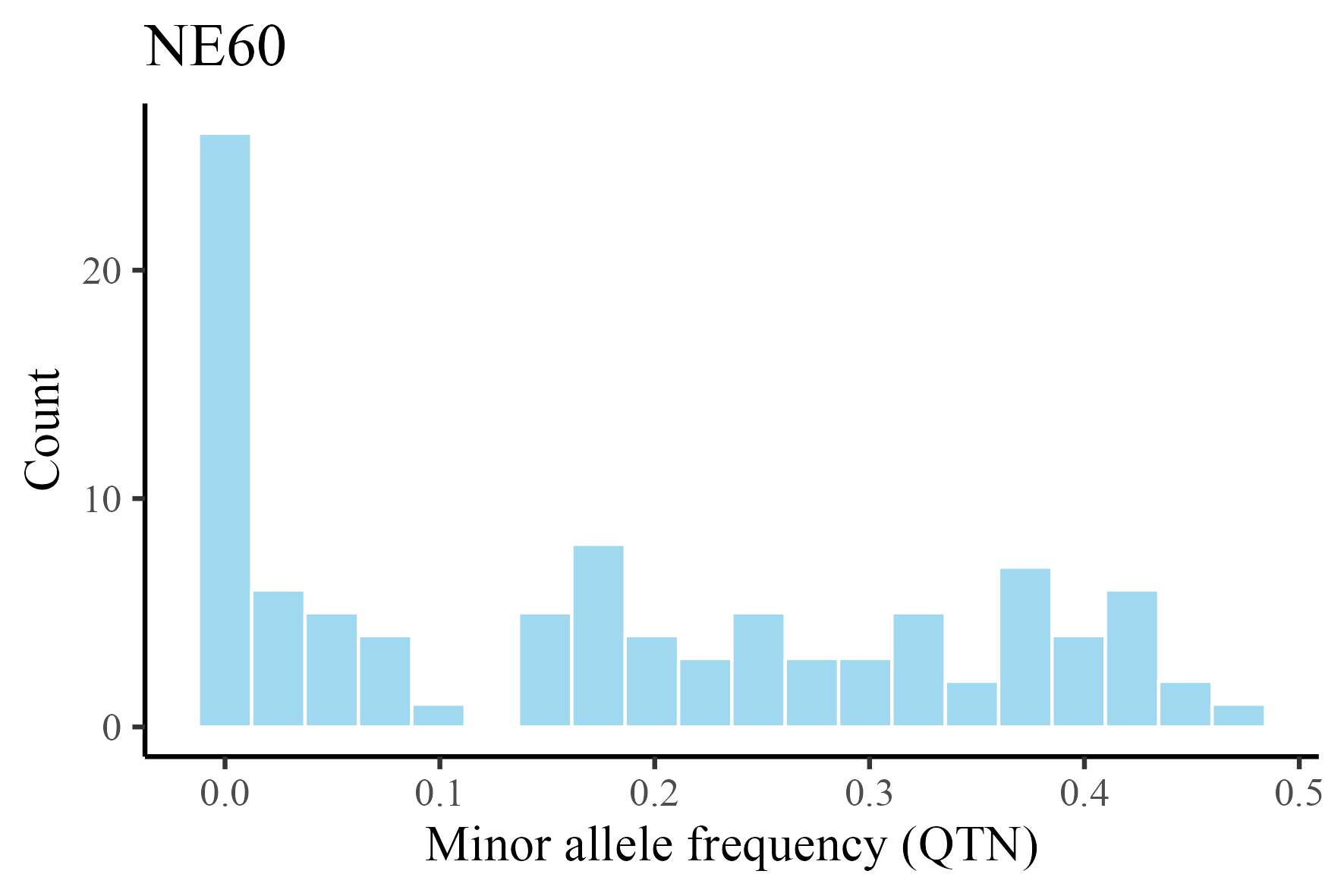


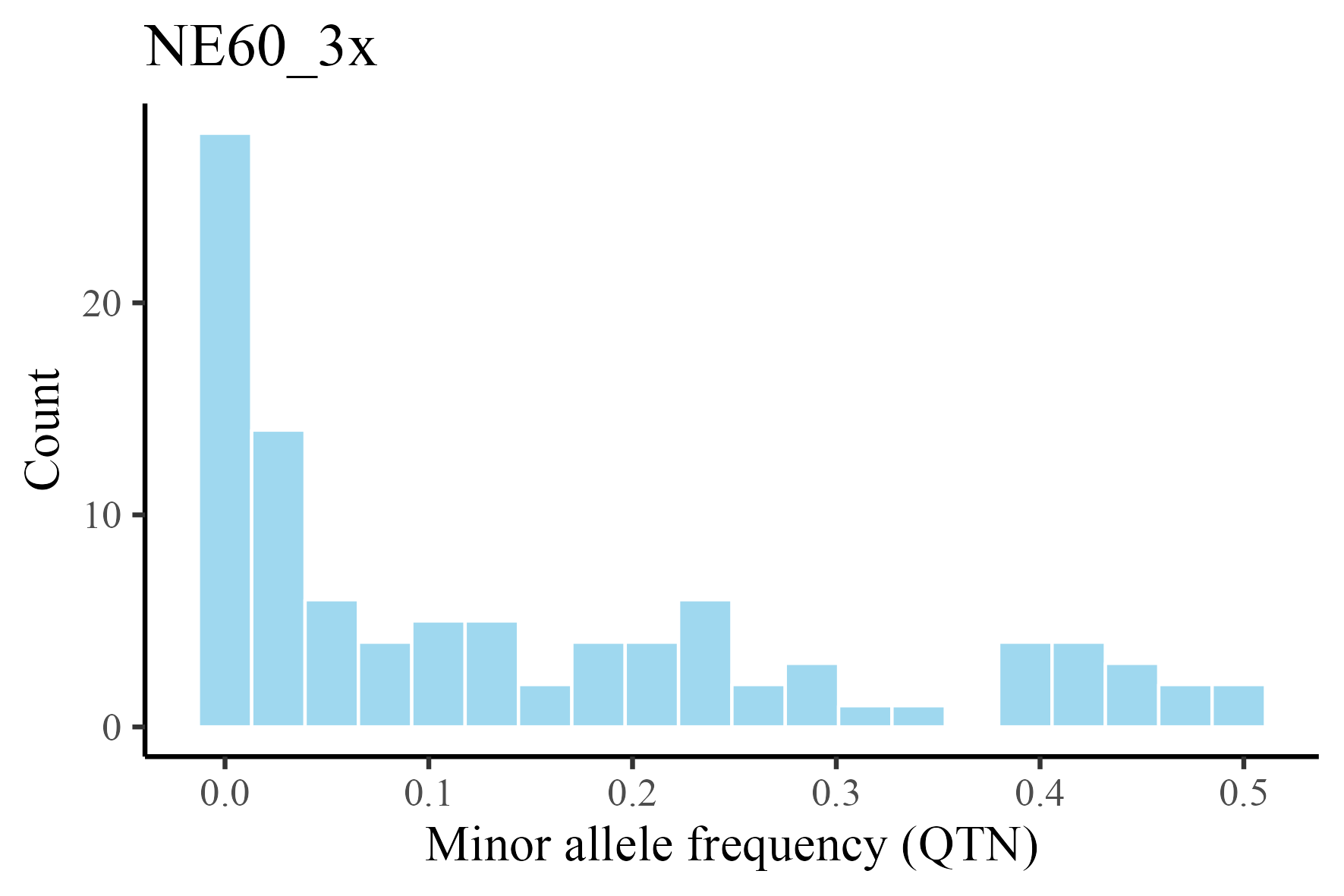


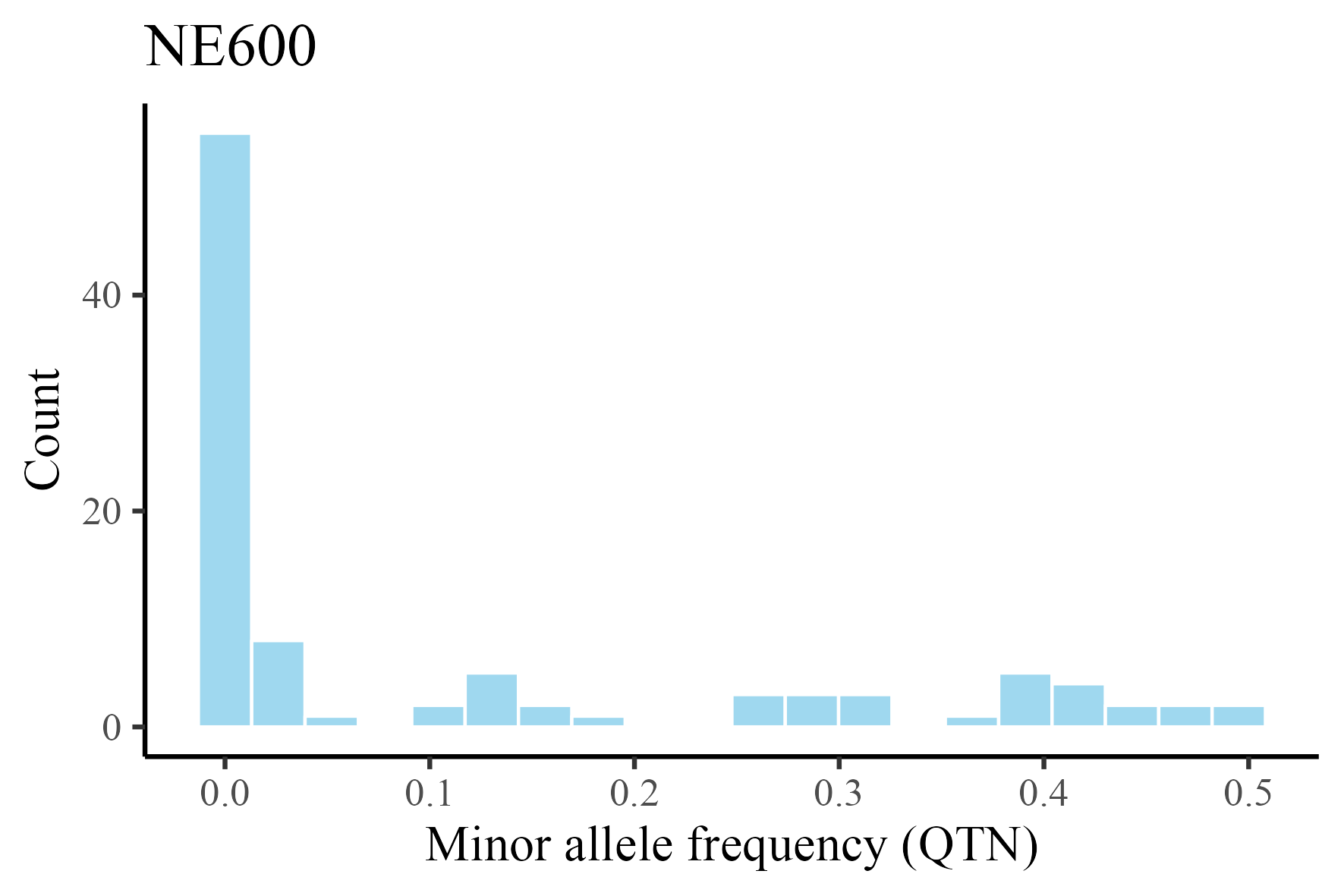

Supplement: iyae103_Supplementary_Data [file iyae103_supplementary_data.zip › Supplemental_File_2_GENETICS-2024-307006.docx]
