# Supplementary material for: Single nucleotide polymorphism profile for quantitative trait nucleotide in populations with small effective size and its impact on mapping and genomic predictions
Source: Genetics. 2024 Jun 24;227(4):iyae103. doi: 10.1093/genetics/iyae103 (PMC11304960; doi:10.1093/genetics/iyae103)

**Supplemental File 3.** Minor allele frequencies for simulated SNP markers that harbor QTN plotted against corresponding estimated SNP effects (absolute value) for a dataset with effective population size 60 (NE60) across 10 chromosomes.


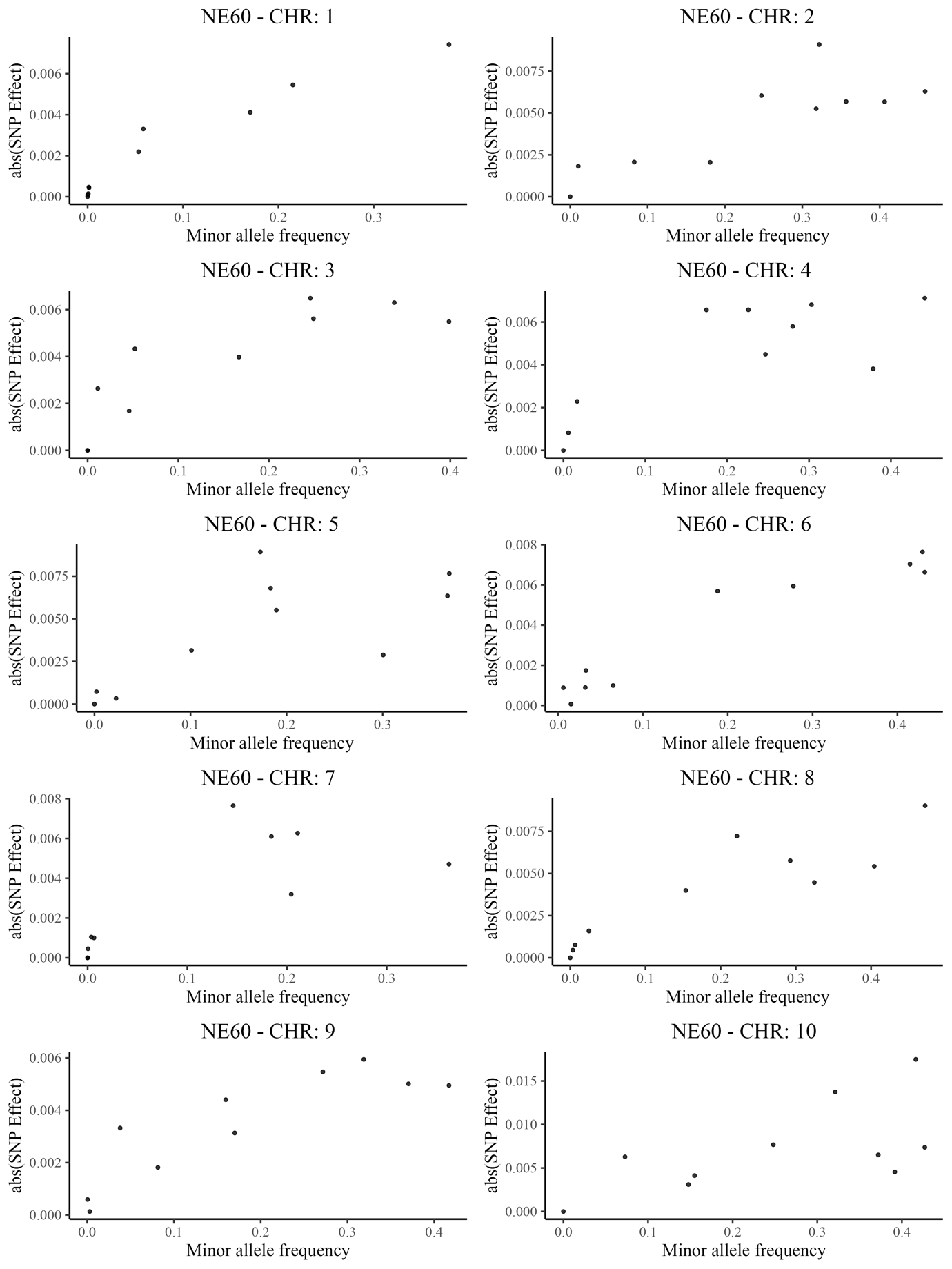

Supplement: iyae103_Supplementary_Data [file iyae103_supplementary_data.zip › Supplemental_File_3_GENETICS-2024-307006.docx]
