# Supplementary material for: Single nucleotide polymorphism profile for quantitative trait nucleotide in populations with small effective size and its impact on mapping and genomic predictions
Source: Genetics. 2024 Jun 24;227(4):iyae103. doi: 10.1093/genetics/iyae103 (PMC11304960; doi:10.1093/genetics/iyae103)

**Supplemental File 5.** Profile of QTN or distribution of SNP around QTN, for 20 QTN with highest minor allele frequency, computed for datasets with effective population size 60 (NE60), with the same effective population size but 3 times more data (NE60_3x), and with effective population size 600 (NE600). Vertical red lines indicate locations of simulated QTN.


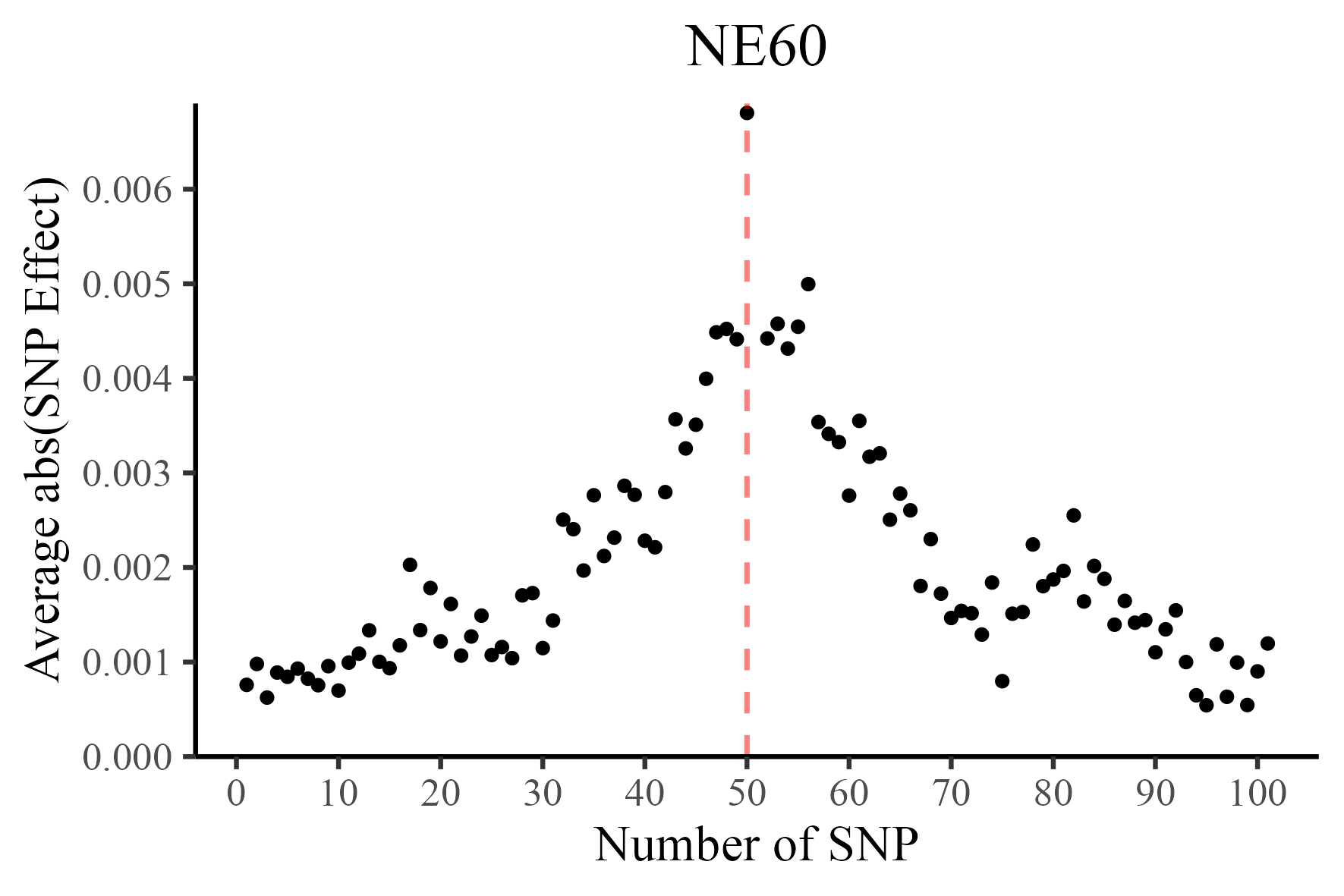

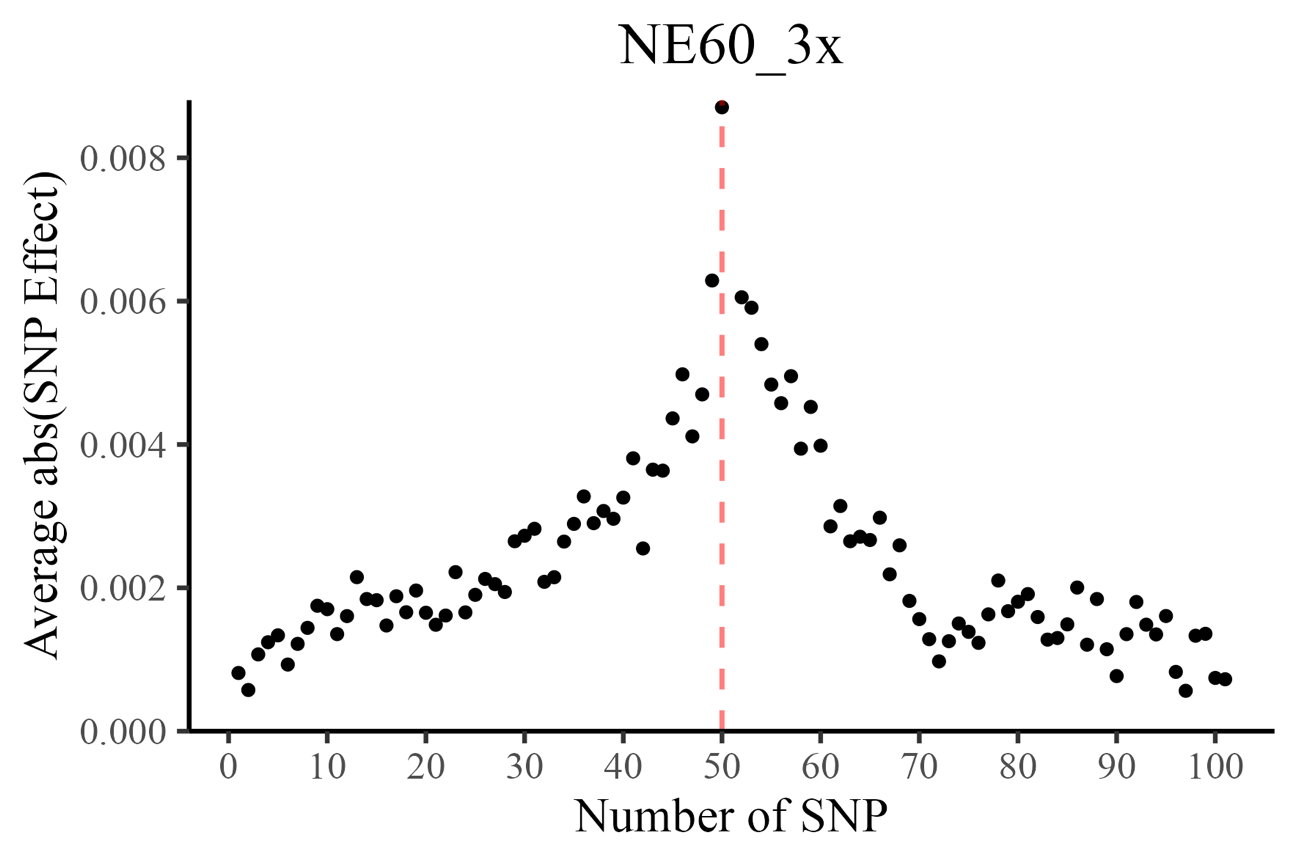


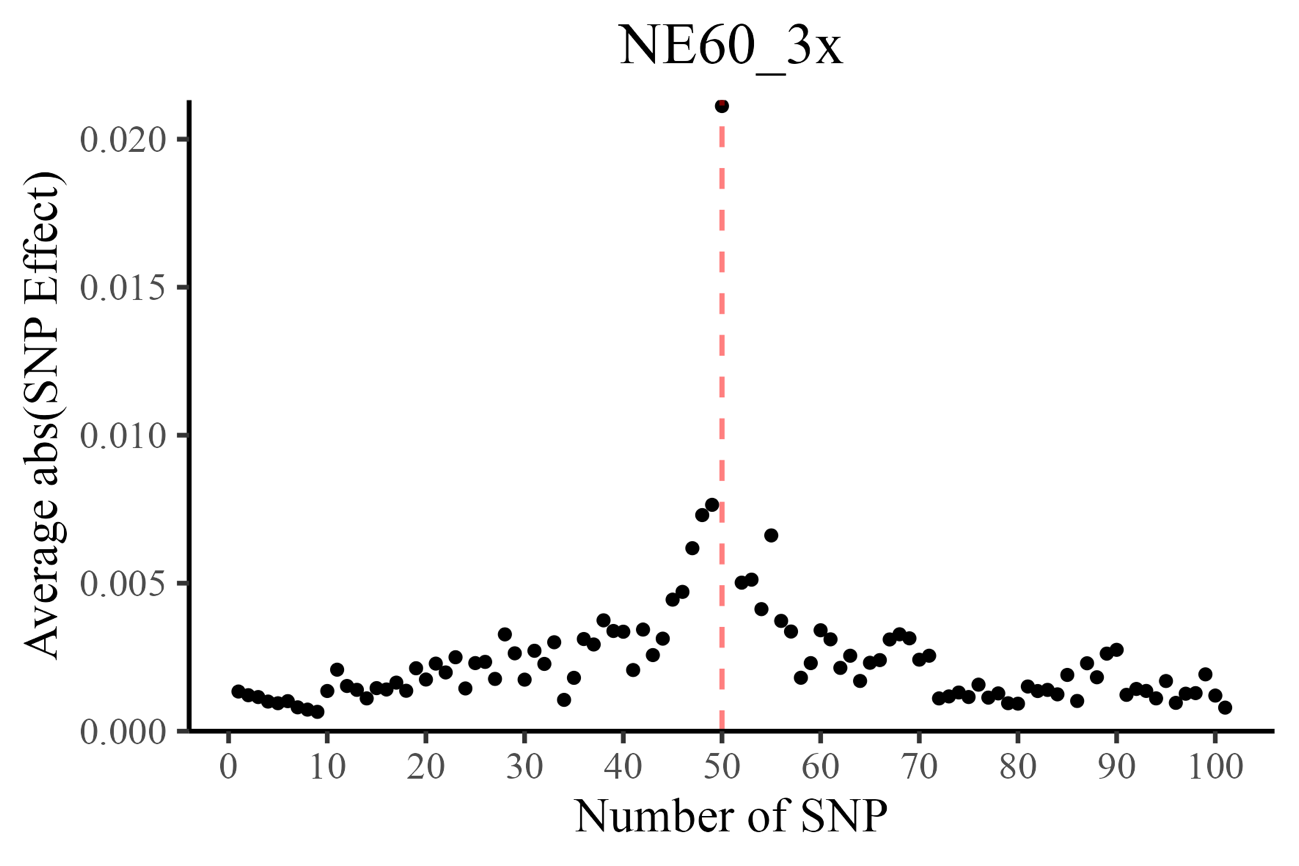

Supplement: iyae103_Supplementary_Data [file iyae103_supplementary_data.zip › Supplemental_File_5_GENETICS-2024-307006.docx]
