# Supplementary figures and images for: Single nucleotide polymorphism profile for quantitative trait nucleotide in populations with small effective size and its impact on mapping and genomic predictions
Source: Genetics. 2024 Jun 24;227(4):iyae103. doi: 10.1093/genetics/iyae103 (PMC11304960; doi:10.1093/genetics/iyae103)

**Supplemental File 4.** Side-by-side Manhattan plots.


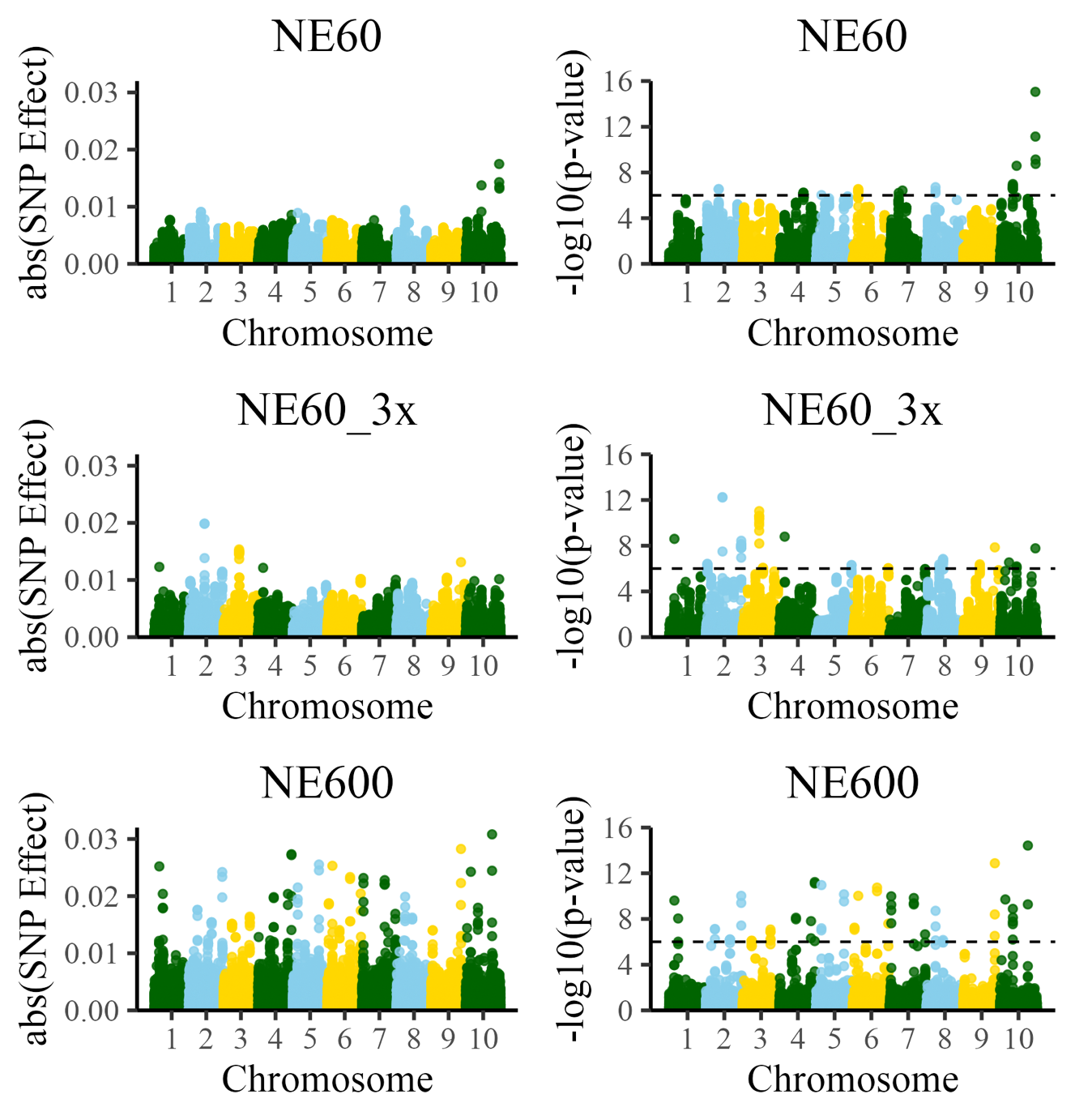

Supplement: iyae103_Supplementary_Data [file iyae103_supplementary_data.zip › Supplemental_File_4_GENETICS-2024-307006.docx]
